# Editorial: Flower Metabolism and Pollinators

**DOI:** 10.3389/fpls.2021.776978

**Published:** 2021-10-06

**Authors:** Monica Borghi, Robert R. Junker, Dani Lucas-Barbosa, Marcin Zych

**Affiliations:** ^1^Department of Biology, Utah State University, Logan, UT, United States; ^2^Department of Biology, Philipps-University Marburg, Marburg, Germany; ^3^Bio-Communication & Ecology Group, ETH Zürich, Zurich, Switzerland; ^4^Vetsuisse and Medical Faculty, National Centre for Vector Entomology, Institute of Parasitology, University of Zürich, Zurich, Switzerland; ^5^Botanic Garden, Faculty of Biology, University of Warsaw, Warsaw, Poland

**Keywords:** bio-communication, chemodiversity, florivory, floral volatiles, folivory, pollination

Outcrossing species that use animals as vectors of pollen transfer lure pollinators by exhibiting floral chemical signals that stimulate their visual, olfactory, and gustatory apparatus. It is said that these traits undergo selective pressure exerted by pollinators according to their specific preferences. However, many of these specialized metabolites initially evolved to protect flowers toward abiotic and biotic stresses and were later co-opted to mediate plant–pollinator interactions. This Research Topic collects recent advances on the chemistry of floral traits, providing insights on the chemodiversity of scent composition and pigments, the intrinsic genetic factors controlling their phenotypical manifestation, as well as the climatic and biotic factors which influence them.

Coevolution between plants and insects is associated with increased biodiversity and both mutualistic and antagonistic interactions contribute to diversification of traits and species. Eilers et al. characterized a remarkable intraspecific variability in flower production, floral metabolic composition and pollen quality in *Tanacetum vulgare*, which affected floral attractiveness for florivorous beetles. Interestingly, not only the chemical phenotype of individuals determined the interaction frequency between the flowers and the antagonists, but also the chemotype of neighboring *T. vulgare* individuals contributed to the susceptibility to florivory. Powers et al. recorded the timing of floral scent emission of two endemic Hawaiian plant species, *Schiedea kaalae* and *S. hookeri*, that share pollination by an endemic Hawaiian moth. While the scent of both species bouquet differed in scent compounds, they shared the timing of peak emissions of floral scent, which coincided with the activity of the moth pollinator. Salzman et al. demonstrated that volatile compounds produced by closely related species of *Zamia* cycads are strikingly different from each other, and that two distantly related pollinating weevil species respond specifically to volatiles from their host Zamia species. The authors highlight chemical communication as a key mechanism of coevolution between cycads and their weevil pollinators. Braunschmid and Dötterl were interested in whether rarity in floral scent related to higher pollination success and fitness. They studied two populations of the deceptive orchid *Cypripedium calceolus* and tested whether flowers with rarer scent bouquets within these populations had more pollination success than flowers with more common scents. The authors found that rarity in floral scent was not correlated with the prospect of the plants setting fruits.

The studies of Sundaramoorthy et al. and Han et al. focus on the genetic and cellular mechanisms underlying the display of floral pigmentation and their spontaneous fading. Using soybean flowers as a model for anthocyanin biosynthesis, Sundaramoorthy et al. identified four recessive purple-blue EMS-induced mutants that all had increased pH in their petals compared to wild-type flowers. Via genetic mapping, the authors of this study showed that the MYB transcription factor GmPH4 and the vacuolar P3A-ATPase GmPH5 gene concur to vacuolar pH regulation, therefore, control changes in petal pigmentation from light pink to dark blue-purple. A transcription factor of the MYB family (MhMYB10) was also associated with the petal pigmentation of *Malus halliana*, of which the color spontaneously fades after pollination had occurred. Interestingly, Han et al. observed a high level of DNA methylation in the promoter region of the MhMYB10 gene, which is associated with decreased expression of MhMYB10 and the downregulation of anthocyanin biosynthetic genes. Finally, Liu et al. by surveying twenty different *Brassicaceae* genotypes for the content of their major floral specialized metabolites via liquid chromatography-mass spectrometry (LC-MS), identified the metabolic features which better discriminate self-compatible (SC) from self-incompatible (SI) species. In particular, the authors identified phenylacylated-flavonoid, and five phenolamides were indicated as significant contributors to this discrimination providing new insights on floral specialized metabolism in relation to the environment and their divergent evolution under biotic/abiotic stresses.

Changes in land use alters habitat affecting the community composition of plants and their pests, along with beneficial insects such as pollinators and predators of herbivorous pests. Landscapes dominated by agriculture are frequently associated with lower diversity of pollinators and higher susceptibility to herbivore pests. Schroeder et al. synthesize evidence of changes in plant trait across land use gradients and discuss the potential for plant adaptation across agricultural landscapes. Their data from a common garden experiment on three wild *Brassicaceae* suggests variation in defensive and reproductive traits along an agricultural gradient. Climate change is also posing an increasing challenge for stability of plant-pollinator interaction. Höfer et al. investigated links between flower visitor behavior and floral traits in the context of increasing drought and temperature and found that the short-term water stress does not alter initial attraction of *Sinapis arvensis* flowers but negatively impacts bumblebees' visitation on flowers resulting in their inferior pollination services. Farré-Armengol et al. quantitatively summarize data on floral-scent emissions from more than 300 plant species exposed to adverse climatic conditions and further discussed implications for pollinators. Finally, Ruiz-Hernández et al. investigate the effect of human selection and breeding on floral traits of ornamental snapdragon plants discovering that human and bee preferences align well for color and scent of the parental lines.

This Research Topic covers various aspects of flower metabolism that are of relevance for the interaction with animal pollinators and provide insights on plant-pollinator communication mediated by floral chemical compounds, and the consequences for the evolution of floral traits, across natural and human-modified habitats.

## Author Contributions

All authors listed have made a substantial, direct and intellectual contribution to the work, and approved it for publication.

## Conflict of Interest

The authors declare that the research was conducted in the absence of any commercial or financial relationships that could be construed as a potential conflict of interest.

## Publisher's Note

All claims expressed in this article are solely those of the authors and do not necessarily represent those of their affiliated organizations, or those of the publisher, the editors and the reviewers. Any product that may be evaluated in this article, or claim that may be made by its manufacturer, is not guaranteed or endorsed by the publisher.

